# Dual-Task Effects on Performance of Gait and Balance in People with Knee Pain: A Systematic Scoping Review

**DOI:** 10.3390/jcm9051554

**Published:** 2020-05-21

**Authors:** Rula Abdallat, Feras Sharouf, Kate Button, Mohammad Al-Amri

**Affiliations:** 1 Department of Biomedical Engineering, Faculty of Engineering, The Hashemite University, P.O. Box 330127, Zarqa 13115, Jordan; rulag@hu.edu.jo; 2 Brain Repair & Intracranial Neurotherapeutics (BRAIN) Unit, School of Medicine, Cardiff University, Cardiff CF24 4HQ, UK; SharoufFH@cardiff.ac.uk; 3 School of Healthcare Sciences, College of Biomedical and Life Sciences, Cardiff University, Cardiff CF24 0AB, Wales, UK; ButtonK@cardiff.ac.uk; 4 Biomechanics and Bioengineering Centre Versus Arthritis, Cardiff University, Cardiff CF10 3AX, Wales, UK

**Keywords:** anterior cruciate ligament, osteoarthritis, knee pain, dual-task, gait, balance, cognition

## Abstract

Dual-task paradigms have been increasingly used to assess the interaction between cognitive demands and the control of balance and gait. The interaction between functional and cognitive demands can alter movement patterns and increase knee instability in individuals with knee conditions, such as knee anterior cruciate ligament (ACL) injury or osteoarthritis (OA). However, there is no consensus on the effects of dual-task on gait mechanics and balance in those individuals. This systematic scoping review aims to examine the impact of dual-task gait and standing balance on motor and cognitive performance in individuals with knee OA or ACL injury. A comprehensive search of MEDLINE, PubMed, Web of Science, and EMBASE electronic databases up until December 2019 was carried out. Inclusion criteria was limited to include dual-task studies that combined cognitive tasks performed simultaneously with gait or standing balance in individuals with knee OA or ACL injuries. In total, fifteen studies met the inclusion criteria, nine articles examined dual-task effects on balance, and six articles reported the effects of dual-task on gait. The total number of individuals included was 230 individuals with ACL injuries, and 168 individuals with knee OA. A decline in gait and balance performance during dual-task testing is present among individuals with ACL injury and/or ACL reconstruction and knee OA. Further research is required, but dual taking assessment could potentially be used to identify individuals at risk of falling or further injury and could be used to develop targeted rehabilitation protocols. A variety of outcome measures have been used across the studies included, making comparisons difficult. The authors, therefore, recommend developing a standardized set of biomechanical balance variables.

## 1. Introduction

Traditionally, motor activities such as gait were thought to be a series of automated movements, requiring minimum cognitive demands [1]. However, many studies [2,3,4] have challenged this notion and established that there is a relationship between motor performance and cognition. A purposeful locomotion requires the ability to adjust to overcome environmental burdens, meet goals, and involves performing a concurrent cognitive task while performing a motor task. Such interaction can be explored using dual-task methodology. Dual-task is used to assess the simultaneous performance of a postural task and a cognitive task or motor task to study the interaction or the effect of the secondary task on the performance of the primary task [5]. Dual-task interference occurs due to the conflict arising in the allocation of attention between the two tasks [6]. 

Dual-task interference has been explored using dual-task paradigms that represent a growing area of research. The effect of cognitive demands on physical performance has been studied for different populations, such as those suffering from traumatic brain injuries [7], strokes [8], Parkinson’s disease [9], Alzheimer’s disease [10], and healthy individuals [11,12]. It is thought to be attenuated in the elderly population due to an age-related decline in the control of input processes [13], decreased ability of task automation [14], or a reduction in time-sharing at the response-selection stage of processing [15]. Despite this growing research interest in the dual-tasking effect, the relationship between cognition and different motor tasks is not clearly established with individuals suffering from knee pain and, in particular, knee OA and ACL injury. 

Rupture and reconstruction of the ACL and knee osteoarthritis are amongst the most common knee conditions. The prevalence of knee osteoarthritis in the United States, for example, is that there is around 14 million people with symptoms of knee OA, and with half of this number being less than 65 years old [16]. The incidence of ACL rupture is not clearly reported, but it is estimated to affect between 30 and 78 per 100,000 people; reconstruction interventions are reported to be around 50–70% of the injured population [17,18]. Knee pain is usually attributed to degenerative changes in the knee joint [19,20,21], such as knee osteoarthritis (OA), which is an age-related disease and is considered one of the most common musculoskeletal disorders worldwide [22]. However, knee OA is not limited to the elderly population; it can develop following anterior cruciate ligament (ACL) injury [23], commonly found amongst athletes. A torn ACL rarely heals or returns to its original anatomic and physiological state. Therefore, untreated ACL injury increases the risk of developing knee OA [24,25]. Attempts to reconstruct ACL, however, fails to restore normal kinematics of the knee joint. Reconstructed knees have a greater incidence and severity of OA than the contralateral non-reconstructed knees, which implies degenerative changes are secondary to ACL rupture and reconstruction [26]. Knee pain, on the other hand, is a common clinical symptom in adult population [27], with nearly 50% of adults over the age of 50 reporting chronic symptoms [20]. It contributes greatly to physical and functional disability [28], leading to increased risk of falling, loss of balance, and increased reliance on cognitive sources to compensate for loss of control [11,28,29]. Consequently, knee pain reduces the ability to perform motor tasks while performing another task in elderly populations [30,31]. 

Given the fact that OA has no permanent cure, with the affected population mainly relying on conservative management protocols that involve physical activities [32]. Dual-task paradigms are needed to implement better therapy protocols and to improve understanding of underlying mechanisms for such disorders. The aim of this review was to assess the effect of cognitive interference on motor activities during walking and standing balance, utilizing dual-task (cognitive task with motor task) paradigms for subjects suffering from knee OA or ACL injury or reconstruction. The review was undertaken to achieve better understanding of the interaction between cognitive demands and motor tasks, which is beneficial to plan future trials and clinical decision making for better therapy programs.

## 2. Methods

In line with methodological guidance of scoping reviews [33], a comprehensive search in the MEDLINE, PubMed, Web of Science, and EMBASE databases was conducted, combining search terms to capture the relevant literature published from the database’s inception until December 2019. Search terms included: Dual-task/tasking and knee, Dual-task/tasking and knee pain, Dual-task/tasking and ACL, Dual-task/tasking and osteoarthritis, cognitive challenge and knee, and cognitive challenge and knee pain. The following formula using Boolean operators was used: (“dual-task” OR “dual task” OR “dual tasking” OR “attention” OR “cognition”) AND (“balance” OR “sway” OR “kinetics” OR “kinematics” OR “gait” OR “postural control”). Then the search was combined using the AND operator with populations of interest: (“anterior cruciate ligament” OR “ACL”), (“knee osteoarthritis “OR “knee OA” OR “knee pain”), the search was limited to human subjects and English language. The reference lists of included articles were manually searched to identify other related articles for inclusion. Inclusion criteria were: (a) Investigates a dual-task that combined a cognitive task with walking or standing; (b) compared the dual-task to single task walking or balance on one or two legs; (c) outcome measures of gait parameters, balance performance, and other measures of functional performance must be reported; (d) population studied was knee pain, ACL reconstruction or deficiency, or knee OA; (e) research designs: randomized and non-randomized controlled trials, pre and post interventions studies, case-control studies, and case studies and reliability studies. 

Two reviewers (M.A.A. & R.A.) independently screened the titles and abstracts to assess if studies were eligible. For those that met the eligibility criteria, the full text was retrieved. Studies that did not meet the inclusion criteria were excluded. Data extracted from the eligible articles were: study and sample characteristics, motor and cognitive tasks characteristics, and findings. This was completed by the first reviewer (R.A.) and checked by the other reviewers (F.S., M.A.A., and K.B.). Data were extracted onto a standardized form and reported narratively.

## 3. Results

**Figure 1:**
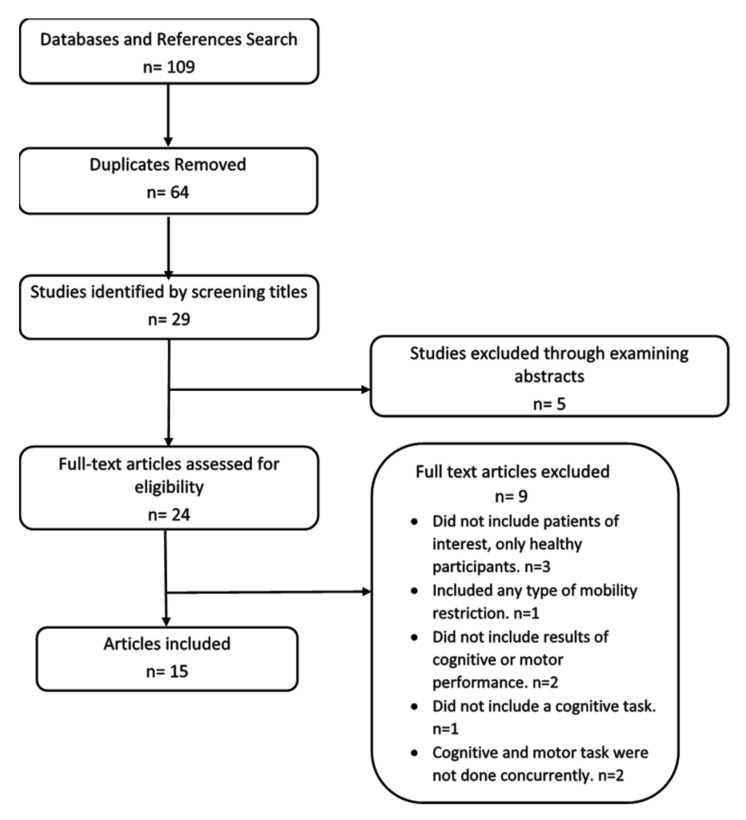
Flowchart showing the process of study selection.

The search yielded 109 articles, and after removing duplicates and screening titles, the abstracts of 29 articles were assessed. Of these, the full text manuscript was retrieved for 24 studies. After reviewing these against the inclusion criteria, 15 articles were included in the evaluation and synthesis (Figure 1). There were 9 studies concerning cognitive dual-task and standing balance (6 ACL and 3 OA) and 6 studies for cognitive dual-task and gait (3 ACL and 3 OA). Data on the study characteristics and findings for cognitive dual-tasking and standing are presented in Table 1 and cognitive dual-tasking and walking are in Table 2. Of the 15 studies, one was a randomized controlled study, while the rest were case-control or case studies.

### 3.1. Dual-Task Effects on Standing Balance for Subjects with Knee OA

Three case-control studies [34,35,36] explored the dual-task effects of a cognitive task on balance control in individuals with OA compared to healthy subjects. In total, 76 subjects with knee OA were included. The study findings are summarized in Table 1a. Two of the studies examined the interaction between cognitive challenge (silent backward counting) and postural stability whilst double leg standing under a variety of conditions (rigid surface/open and closed eyes, and foam surface open/closed eyes) [34,35]. The third study [36] looked at recovering standing balance to forward induced falls whilst performing a cognitive challenge (backward counting).

In all of these studies, performing a cognitive task whilst concurrently performing a balance activity resulted in a statistically significant reduction in the magnitude of the balance outcomes in people with knee OA and healthy participants. Also, there was a significant difference between the healthy and OA participant groups in general, but no interaction between groups and balance condition.

### 3.2. Dual-Task Effects on Standing Balance for Subjects with ACL Injuries or Reconstruction

In total, six studies evaluated the use of dual-task on posture control and stability for ACL deficient and reconstructed subjects during single and double leg standing balance, across a variety of different dual-task paradigms, with different balance outcomes. The characteristics of these studies and findings are presented in Table 1b. One study [37] assessed the ability of individuals with ACL injury (deficiency and reconstruction) and healthy controls to maintain single leg balance whilst performing a simultaneous cognitive task (Auditory Stroop test) when using a SMART EquiTest dynamic balance system board, which moved in a forward and backward direction. The main finding was that a significant reduction in reaction time was reported during large cognitive disturbance for ACLR subjects only, while no significant difference was noticed in the ACLD population 

The contribution of attention to postural control in 54 subjects with ACL injury and a matching number of healthy controls was evaluated in two studies [38,39]. Subjects were asked to perform a cognitive task (backward digit span test) while standing on a force plate in two different conditions (double limb stance (DLS), and single limb stance (SLS) of preferred choice, i.e., dominant leg). The postural task was altered by changing the characteristics of the surface where subjects stood on (Rigid/Foam surface). In both studies, both groups showed reduced center of pressure regularity when moving from single to dual-tasks conditions. Introducing a cognitive task had no significant effect on postural stability in subjects with ACL deficiency compared to healthy controls for DLS or SLS conditions on the rigid and foam surfaces. 

Three studies assessed the effect cognitive demands on postural balance and stability on 61 ACLR subjects. One study [40] tested the effect of silent backward counting on DLS balance for 19 ACLR subjects and 21 healthy controls. The postural task involved standing on a force platform at different conditions (Foam/rigid surface, open/closed eyes). No significant difference in sway measures for ACLR was noted in comparison to healthy participants across the conditions studied. 

The effects of cognitive demands on postural stability for ACLR and a matching control groups were examined during SLS using an instrumented wobble board [41] and the Biodex balance system [42], both of which are dynamic posture assessment tools. The effect of the cognitive task was significant in both studies. The ACLR group showed significantly higher contact frequency and longer contact time in [41], whereas in [42], a sacrificed cognitive performance, higher values of overall stability index, and anterior-posteriors stability index were shown. 

Four studies evaluated the ability of ACL to maintain balance during SLS only, where two of them [37,42] tested balance when subjects were standing on their injured limb, and the other two studies [39,41] looked into balance differences in SLS when standing on the injured/uninjured limb. One study assessed balance during DLS only [40], and another study looked at balance in the presence of a cognitive task in both DLS and SLS [38]. Only three studies reported significant dual-task interaction; this was reported in terms of decreased postural stability [41], a sacrificed cognitive performance to gain safe balance [42], or a significant decrease in reaction times during complex cognitive tasks [37]. 

### 3.3. Dual-Task Effect on Walking for Populations with Knee OA

Three studies looked at the effects of dual-task on gait performance for populations with knee OA. One was a randomized controlled trial [43], the other two [44,45] were pre-post intervention studies. A total of 92 knee OA subjects were included. The findings are summarized in Table 2a. 

The effects of a walking exercise program on dual-task performance on 40 females suffering from knee OA was done in [43], participants were randomly divided into a walking group and a control group. Both groups had a four weeks physical intervention program with the walking group and were asked to increase the number of steps daily performed. The ability to perform dual-tasks was examined for both groups after four weeks. Subjects were asked to walk at a self-selected speed on a 16-m-long pathway alone or while doing three serial subtractions as a cognitive task. Different walking parameters such as automaticity, trail making test performance (∆TMT), and functional abilities of knee parameters before and after invention were measured. The other study [44] assessed the influence of pain on cognitive functions and executive control in 36 patients with knee OA before and after knee replacement. Subjects were asked to walk on an 18-m pathway with preferred walking speed with and without performing a secondary cognitive task (three serial subtractions) at two different sessions, the first one prior knee replacement surgery and the other 6–8 weeks after performing the surgery. Sensors attached to participant’s feet were used to collect kinematic gait series, and then used to measure stride to stride variability using coefficient of variation of minimum toe clearance; the dual-task costs were calculated based on percentage change of gait variability between single and dual-task, and the correlation between pain severity and DTC was explored. 

The last study [45] evaluated the ability to learn to change the foot progression angle following a six week training program in subjects with knee OA. A total of 16 knee subjects with OA underwent a six week-long training program, in which participants were asked to walk on a treadmill with a preferred walking speed while their lower limb and trunk data were collected by a motion capture system to calculate foot progression angle and knee abduction moments in real time. Training time was increased gradually from week 1 to week 5 by 3 min. During the first and the last week, participant foot progression angle difference was assessed in four conditions performed at a specific order (natural walking, walking with feedback on foot progression angle, walking without feedback, and dual-task walking without feedback). The cognitive challenge of choice was a visual Stroop test, where participants are required to verbally respond to the color of the word that appear on a screen while walking. Mean foot progression angle was calculated across stance phase and compared to the target value in term of the difference between obtained and target.

In summary, results from all three studies reported significant dual-task interference between cognitive loading and motor tasks in subjects with knee OA represented by a change in dual-task costs. Training and exercise decreased dual-task costs shown by an increase in automaticity [43], and a decrease in cognitive loading [43,45]. Pain was shown to have disruptive effects on dual-task performance, as pain reduction was related to decreased dual-task costs shown by a decrease in gait variability when comparing single and dual-task conditions in knee OA subjects [44].

### 3.4. Dual-Task Effect on Walking for Subjects with ACL Injuries or Reconstruction

Only three studies looked at the effects of dual-task on walking for subjects with ACL deficiency (ACLD) [46,47] or post reconstruction (ACLR) [48]. One study investigated the use of dual-task in assessing inter limb differences in subjects who had ACL reconstruction [48]. The other two [46,47] were case-control studies focusing on the effects of cognitive loading on gait measurements for ACL groups and controls during walking. In total, 42 ACLD, 25 ACLR, and 42 healthy participants were recruited across the three studies. The studies used varied postural and cognitive tasks to assess the effects of walking speed, base of support, and cognitive loading on subjects with ACLD and ACLR.

One study investigated [46] the effects of dual-task on the variability of stride to stride for 22 ACLD subjects and a matching control number. Participants were asked to walk on treadmill at three different velocities (high, low, self-selected) while performing a concurrent cognitive task (Auditory Stroop Test). A motion capture system was used to record and analyze gait for the dominant leg of the ACLD group and the matching limb in the control group. The knee flexion-extension Lyapunov exponent (LyE) was utilized to find the sagittal angular knee displacement in time series. Reaction time was also calculated to measure the cognitive performance.

Another study [47] looked at the effects of narrow base walking and dual-task on gait. Twenty individuals with ACLD and a matching control group were asked to walk on a path with two base of support conditions (normal/narrow) while performing a concurrent cognitive task (Backward counting). Gait patterns involving the mean and variability of step velocity and length were assessed during a single and dual-task condition for the two base of support conditions. 

Only one study involved individuals with ACLR [48], which examined the effect of dual-task walking on different gait parameters in ACLR. Twenty-five participants with ACLR were asked to walk along a 10-m walkway at self-selected speed with and without a concurrent cognitive task (backward counting). Different kinematics and kinetics parameters of the lower extremity were measured using motion capture system and two embedded force plates in the walkway. All three studies reported significant dual-task interference on gait parameters. This was shown in terms of a sacrificed Cognitive performance for individuals with ACLD to maintain safe gait [46], a reduction in step velocity in dual-task conditions for individuals with ACL when walking on a narrow base of support [47], and a significant decrease in inter-limb differences in the presence of cognitive demand [48].

## 4. Discussion

This is the first systematic scoping review assessing the effects of dual-task paradigms combining a cognitive task with gait and balance tasks in individuals with knee OA or ACL injury and/or reconstruction. These findings can be used to improve the rehabilitation protocols for these individuals. Our review findings suggest that there is a body of evidence on the effects of cognitive tasks on motor performance in patients with knee conditions. Sixty percent of the evaluated studies showed a significant group by task interaction effect of a concurrent cognitive task on balance or gait [37,41,42,43,44,45,46,48]. It is worth saying that a mixed response of dual-task interference was observed, and gait seemed to be more affected than balance (six gait studies vs. 3 balance studies), which might be related to the fact that gait, unlike balance, requires more cognitive resources. Our findings agree with a recent review [49], which examined the effects of cognitive loading on motor performance in injured individuals suffering from ankle, knee, and low back injuries, in comparison to healthy controls. However, studies on individuals with knee OA were not included despite the large numbers of sufferers. In addition, some reviewed articles looked at the effects of cognitive loading on motor behavior even if the cognitive task was not performed concurrently with the motor task [47]. Their findings suggest that motor performance deteriorates when a cognitive load is introduced concurrently to performing a motor task.

This demonstrates that an assessment using a dual-task paradigm may be able to distinguish between individuals with knee conditions, and give an explanation about the increasing risk of falls and injuries through poor balance in daily life activities in which increased cognitive demands are encountered. Hence, better rehabilitation protocols can be implemented. However, some limitations were encountered within the literature that can affect the translation of these findings into clinical practice. These include small sample size [43], gender selection [46,47], and types and degrees of OA and ACL pathologies in individuals included in these studies [34,38], as well as the lack of uniformity and standardization in the used testing protocols. Moreover, as OA is usually common amongst the elderly, and normal aging is related to decline in cognitive and physical performance, tools such as mental tests, or even using functional magnetic resonance imaging or a psychological refractory period test, can provide an insight into brain activity and age-dependent variations in dual-tasking.

Results obtained from studies concerning individuals with knee OA during standing balance did not show a significant difference of the affected population (i.e., knee OA) more than the healthy controls. Results indicate that a concurrent cognitive task during double and single leg stance has reduced balance and stability for both knee OA and healthy individuals. Only one study [36] reported an increased number of recovery steps to regain balance in knee OA more than healthy individuals, suggesting an increased risk of falling. 

For individuals with ACL pathology while standing, dual-task interference did not show any significant effect on postural balance and stability in comparison to healthy subjects except in three studies [37,41,42] where significant differences between reconstructed ACL and healthy were encountered. Outcomes of the studies concerned with subjects with ACLD showed a deterioration in motor performance for all participants, but there was no interaction between dual-task and populations with ACLD in comparison to healthy controls. Results reported great variability of measured parameters and results. Out of six studies, only three studies reported significant dual-task interaction, which was reported in terms of decreased postural stability [41] and a sacrificed cognitive performance to gain safe balance [42], or a significant decrease in reaction times during complex cognitive tasks [37]. 

The results of the remaining studies did not support the hypothesis made that dual-task interference affects balance and stability for ACL injuries [38,39,40]. For example, two studies [38,39] showed that postural balance was compromised in the presence of a cognitive task, but the cognitive task did not affect postural control in patients with ACL injury more than healthy participants. One study [37] looked at the reliability of proposed protocols to measure dual-task costs or dual-task interference in subjects with ACLD and ACLR, and reported a significant increment in reaction time during complex cognitive tasks in subjects with ACLR. Lion et al. [40] did not show a significant impact of cognitive demands on postural control for ACLR groups when compared to the matching control group in double limb stance. This could be explained by the fact that quiet standing is a well learned static position, which might not be sensitive enough to measure dual-task interference in individuals with ACL injury.

Despite the limited number of studies concerned with knee OA during walking, all the studies showed that physical interventions, such as exercise and retaining normal physiological functions using surgical intervention, reduced dual-task costs. In one of the studies [43], significant improvement in walking parameters for the walking group were noticed while no significant difference was found in the control group, indicating that exercise contributed in the improvement of cognitive functions. Moreover, a decrease in pain severity [44] was related to a reduction in dual-task cost values, implying that such a decrease would reduce tripping risks, and that pain has disruptive effects on motor-cognitive interaction. Richards et al. [45] reported a significant decrease in foot progression angle values. Foot progression angle difference decreased significantly at week 6 when compared to values obtained at week 1, adding feedback that reduced the difference of the foot progression angle to about zero. The interference of a cognitive task on foot progression angle values also showed a decrease between week 1 and 6. 

Similarly, dual-task interference was greater in individuals with ACL pathology while walking compared to healthy controls in all articles. For example, the reaction time during dual-task was higher in ACLD compared to healthy controls [46]. Moreover, Knee LyE in ACLD significantly decreased as the velocities changed from low to high, while no change was found in the control group. This suggests that performing dual-tasks in ACL deficient might result in shifting the attention to perform safe gait by sacrificing the cognitive task, which indicates a motor-cognition interaction [46]. Inter-limb differences were significantly reduced in ACL reconstructed athletes during dual-task [48], where walking speed decreased significantly when subjects performed a concurrent cognitive task. Mazaheri et al. [47] showed that gait stability and pattern during dual-task for injured subjects was modified under increased visuomotor demands (i.e., narrow Base of Support (BOS)) in comparison to the control group.

Overall, it should be noted that out of 15 studies, one reliability study was conducted to measure the inter- and intra-session reliability of using a dual-task paradigm as a reliable tool for measuring the interaction between physical and cognitive processes [37]. The limited number of studies on populations with knee OA was unexpected, given the fact that OA is an age related disease and many studies have looked into the interaction between physical and cognitive demands in healthy elderly people [2,11,12]. Despite the positive findings reflecting the importance of the interaction between cognitive challenges and physical activities, existing literature remain scarce, especially when compared to the amount of literature studying dual-task effects on other populations. 

Pain accompanied with such injuries and pathologies can affect cognitive performance in negative ways [48,49]; hence, a decline in dual-task performance can be expected in populations with knee conditions as a consequence of pain and injuries. However, it was noticed that studies looking into the effect of dual-task interference on balance reported non-significant effects during standing. This is thought to be due to the fact that standing and balance does not require higher attentional and cognitive demands, and thus, the effect of dual-task was not observed. 

Therefore, the implementation of standardized and well-designed protocols for measuring dual-task interference in future studies are needed to get a better understanding of this topic, given the fact that knee pain and associated injuries are one of the main causes of musculoskeletal disorders. Also, it is recommended to have a standardized set of outcomes to assess balance and gait as a variety of outcome measures were used across the evaluated studies, which would not allow a clear comparison between results. Moreover, results have shown that in double limb stance, the effects of dual-tasks and dual-task costs are harder to detect in patients with knee pain given that the subjects, in general, seem to apply a posture first concept. Hence, more complex and sensitive cognitive and motor tasks are needed to observe dual-task interference and costs. A better understanding of the effects of dual-task on physical as well as cognitive performance would allow for the implementation of better rehabilitation programs, as well as being of vital importance in predicting the initiation of knee degradation process.

Despite the scarcity of studies, this review has established the importance of dual-task paradigms in understanding the effect of pain on motor performance in patients with knee pain, indicating that many of those patients do not seem to have the cognitive capacity to handle dual-task conditions without altering either cognitive or motor performance. Those individuals are thought to walk with more compensatory executive control and less automaticity [50]. The impact of dual-task paradigms on motor performance could be an attractive research topic for future studies. Interestingly, a growing body of literature suggests that dual-task paradigms can have an impact on motor performance and ultimately better rehabilitation programs. For example, the introduction of cognitive dual-task was found to affect motor performance in individuals with chronic lower back pain [51,52]. This is thought to be due to the distracting effect of the dual-task, where task processing and pain processing compete for limited attentional resources described as the attentional-capacity model. According to this model, the complexity of cognitive tasks are considered as critical factors that influence the perceived pain intensity of noxious stimulus; such factors recruit limited attentional resources leaving fewer resources to process pain signals [53]. Dual-task paradigms were tested in other conditions such as Alzheimer’s (AD) [54] and Parkinson’s disease (PD) [5,54]. Dual-task measures appear to evaluate a unique construct in individuals with early to mid-stage in AD and mild-moderate stages in PD, and may have value in improving the prediction of falls risk in this population [54]. Furthermore, dual-task training seems to not be harmful and could be part of the rehabilitation of PD patients [55]. The introduction of such training could lead to the implementation of better rehabilitation programs for individuals with knee pain and should be considered in future studies.

## 5. Conclusions

This review aims to look into the nature and scope of literature studying the effects of dual-tasks on subjects suffering with knee pain. Our findings support the use of dual-task paradigms in assessing functional performance of individuals suffering from knee OA or ACL. The majority of studies that are included in this review established a link between cognitive challenges and physical demands, reporting worse balance and gait parameters with the introduction of a concurrent cognitive task. Subjects with ACL injury or knee OA exhibited significant changes in balance and gait during dual-task in comparison to control groups. Walking and dynamic balance can be considered an effective tool in distinguishing dual-task interference, compared to stand-still balance tasks. Future research should focus on using similar protocols used in studies included in this review and more difficult balance tasks, as well as using verbal cognitive tasks that can assess cognitive abilities. This scoping review also highlights the need to study motor-cognitive interactions in individuals with knee pain. Definitive conclusions cannot be drawn due to the variety of samples, outcome measures, and methodological techniques used. The use of dual-task paradigms has the potential to improve and implement new strategies of rehabilitation programs and different therapy options for those with ACL related syndromes and knee OA. 

## Figures and Tables

**Table 1:** Studies included in this review dealing with dual-task during standing.

Authors + Date	Sample	Postural Task	Cognitive-Task	Aims	Outcomes Measures	Key Findings		
						Group X Task	Group	Task
**Dual-task standing**								
**(a) Knee Osteoarthritis (OA)**								
Negahban et al. (2015) [34] Case control	25 Knee OA 25 Healthy	Double limb stance (DLS) at different conditions: (1) rigid surface–open eyes; (2) rigid surface–closed eyes; (3) foam surface–open eyes; (4) foam surface–closed eyes	Silent Backward Counting	Examine the interaction between posture and cognition in the two groups in terms of **linear sway** measures	Mean velocity, sway area and standard deviation (SD) velocity, and amplitude in AP and ML directions of center of pressure (COP)	Non-significant	Significant Higher SD amplitude and velocity in medial lateral (ML) direction with *p* < 0.05 and higher sway area and mean velocity with *p* < 0.05 in Knee OA compared to healthy	Significant Both groups had decreased sway and less SD amplitude in dual-task conditions
Negahban et al. (2016) [35] Case control	27 Knee OA 27 Healthy	DLS at different conditions: (1) rigid surface–open eyes; (2) rigid surface–closed eyes; (3) foam surface–open eyes; (4) foam surface–closed eyes	Silent Backward Counting	Examine the interaction between posture and cognition in the two groups in terms of **non-linear** sway measures	Central tendency measure (CTM) and percentage of determinism %DET of COP to analyze the variability and complexity of sway	Non-significant	Significant Knee OA had higher %DET and less CTM in ML direction in comparison to healthy controls	Significant All participants had lower %DET and higher CTM in dual-task conditions
Levinger et al. (2016) [36] Case control	24 Knee OA 15 Healthy	Balance after induced falls	Backward counting	Examine balance responses of induced fall conditions during single and dual-task	Spatiotemporal parameters (step length, step velocity, center of mass velocity), and lower limb kinetics and kinematics (lower limb angles, moments, and power)	Non-significantKnee OA had an increased recovery steps number on dual-task	Significant Knee OA patients had a reduced step length, center of mass velocity, hip and knee flexion angles, moments and power, and negative muscle work at knee and ankle joints	Significant Both groups had reduced step length and center of mass velocity in dual-task
**(b) Anterior Cruciate Ligament (ACL) patients**								
Akhbari et al. (2014) [37] Case control	23 ACL deficient (ACLD) 25 ACL reconstructed (ACLR)19 Healthy	Single limb stance (SLS) on (SMART EquiTest) with different difficulty levels	Auditory Stroop test	Determine intra-and intersession reliability of balance and cognitive performance under single and dual-tasks	Reaction time (RT), latency, and amplitude of balance	Not reported Significant difference in reaction time during complex cognitive tasks for ACLR	Not assessed	Not assessed
Negahban et al. (2009) [38] Case control	27 ACLD 27 Healthy	DLS and SLS at different conditions: (1) rigid surface–open eyes; (2) rigid surface–closed eyes; (3) foam surface–closed eyes	Backward Digit Span Test	Study the effect of cognitive load and attentional demands on postural control in ACL deficient patients and healthy control	COP data were collected to measure postural sway variables in AP and ML directions including mean velocity, standard deviation of velocity, and phase plane portrait. Different types of cognitive errors were measured, such as intrusion, omission, and order errors	Non-significant	Significant ACLD had less balance (more sway) and made more cognitive errors than healthy controls	Significant Both groups performed worse and had less balance in dual-task conditions
Negahban et al. (2010) [39]	27 ACLD 27 Healthy	DLS and SLS at different conditions: (1) rigid surface–open eyes; (2) rigid surface–closed eyes; (3) foam surface–closed eyes	Backward Digit Span Test	Study the effects of ACL injury on deterministic pattern of postural sway in single and dual-tasks	%DET and Shannon entropy in AP and ML directions	Non-significant	Significant In DLS, %DET and Shannon entropy was higher in ACLD In SLS, ACLD| had higher %DET and higher Shannon entropy in ML direction	Significant All participants had increased %DET and entropy in dual-task conditions
Lion et al. (2018) [40] Case control	19 ACLR 21 Healthy	DLS at different conditions: (1) rigid surface–open eyes; (2) rigid surface–closed eyes; (3) foam surface–open eyes; (4) foam surface–closed eyes	Silent Backward Counting	Examine the effect of dual-task on postural control for both groups in DLS	Sway area and sway path for center of foot pressure	Non-significant	Non-significant	Significant Both groups swayed less in dual-task
Negahban et al. (2013) [41] Case control	25 ACLR 25 Healthy	SLS on instrumented wobble board (IWB) with different difficulty levels (injured/uninjured) with (straight/flexed) knees.	Silent Backward Counting	Investigate the amount of attentional demands on postural control in the two groups	Contact frequency and contact time	Significant ACLR has higher contact frequency and longer contact time in dual-task compared to single task conditions	Significant ACLR group had longer contact time and higher contact frequency in all conditions compared to healthy controls	Significant Contact frequency and time increased in dual-task for all participants
Mohammadi-rad et al. (2016) [42] Case control	17 ACLR 17 Healthy	SLS on the Biodex Balance system (BBS) with different stability levels (levels 6 & 8) with open/closed eyes	Auditory Stroop Test	Determine the effect of cognitive challenge on dynamic postural stability for the two groups	Overall stability index (OSI), anterior posterior (AP) and ML stability indices (APSI and MLSI)	Significant for OSI and APSI ACLR had worse balance in dual-task condition when performing level 6 with eyes open postural task	Significant in cognitive performance ACLR showed worse cognitive performance than healthy controls for all conditions (longer reaction time and higher error ratio).	Significant for medial lateral stability index (MLSI) Worse balance for all participants in dual-task condition

**Table 2:** Studies included in this review dealing with dual-task during walking.

Authors + Date	Sample	Postural Task	Cognitive-Task	Aims	Outcomes Measures	Key Findings		
						Group X Task	Group	Task
**Dual-Task Walking**								
**(a) Knee OA**								
Hiyama et al. (2011) [43] Randomized controlled trial	40 knee OA females divided into two groups: control and walking groups	Gait Subjects were asked to walk at a self-selected speed on a 16-m-long pathway	Serial Subtraction Test	Study the effect of exercise on dual-task performance	Automaticity index, differences in Trail making test (ΔTMT), and functional abilities measured using Japanese knee OA measure	Significant Walking group had a significant improvement in outcome measures shown by an increase in automaticity, a decrease in ΔTMT, and improved functional abilities in dual-task	Not reported	Not reported Increase in gait time when moving from single to dual-task in all participants
Hamacher et al. (2016) [44] Pre-post	36 knee OA tested pre and post knee replacement surgery	Gait Subjects were asked to walk at a self-selected speed on an 18-m-long pathway	Serial Subtraction Test	Study the effect of pain on dual-task costs (DTC)	Variability of minimum toe clearance in single and dual-task walking Pain severity was measured pre and post operation Dual-task cost was measured as percentage change of gait variability from single task to dual-task	Significant Dual-task costs reduction goes along with pain reduction. Subjects with high differences in pain severity had significant difference in dual-task cost	Not Assessed	Not Assessed
Richards et al. (2018) [45] Pre-post	16 knee OA	Gait Subjects were asked to walk on treadmill	Visual Stroop Test	Study the effect of training on single and dual-task performance	Foot progression angle (FPA)	Significant Further reduction of foot progression angle in dual-task after training.	Not assessed	Significant Foot progression angle decreased when dual-task was introduced
**(b) ACL patients**								
Nazary-Moghadam et al. (2018) [46] Case-control	22 ACLD 22 Healthy	Gait Walk on treadmill at three different velocities (high, low, self-selected)	Auditory Stroop Test	Study the effect of walking speed and cognitive load on stride-to-stride variability	Lyapnov exponent (LyE) was calculated for knee flexion extension to determine the stride to stride variability in single and dual-task. Reaction time and error rate was calculated to measure cognitive performance	Significant for cognitive performance ACLD had higher reaction time during dual-task compared to healthy subjects	Significant at high velocity ACLD had lower knee flexion-extension LyE than healthy	Non-significant
Mazaheri et al. (2016) [47] Case-control	20 ACL 20 Healthy	Gait Walk on a pathway with two base of support conditions(narrow/normal)	Backward Counting	Study the effects of dual-tasking and different base of support on gait	Mean and variability of step length and step velocity	Significant ACL had lower step velocity during dual-task when walking on a narrow base of support	Significant ACL had greater variability for all spatiotemporal parameters	Significant All participants had decreased step length variability in dual-task
Shi et al. (2018) [48] Case study	25 ACLR	Gait walk along a 10-m walkway at self-selected speed	Backward Counting	Study the effect of cognitive task on gait asymmetries in ACLR subjects	Hip and knee angles and moments	Significant Inter-leg difference (ILD) of hip adduction angle and abduction moment decreased in dual-task condition.	Not Assessed	Significant Smaller peak knee flexion angle and extension moment in DT
